# Hypocretin-1 receptors regulate the reinforcing and reward-enhancing effects of cocaine: pharmacological and behavioral genetics evidence

**DOI:** 10.3389/fnbeh.2012.00047

**Published:** 2012-07-24

**Authors:** Jonathan A. Hollander, Don Pham, Christie D. Fowler, Paul J. Kenny

**Affiliations:** ^1^Laboratory of Behavioral and Molecular Neuroscience, Department of Molecular Therapeutics, The Scripps Research Institute, JupiterFL, USA; ^2^Department of Neuroscience, The Scripps Research Institute, JupiterFL, USA

**Keywords:** hypocretin, orexin, cocaine, reward, addiction, intracranial self-stimulation, intravenous self-administration, knockout mice

## Abstract

Considerable evidence suggests that transmission at hypocretin-1 (orexin-1) receptors (Hcrt-R1) plays an important role in the reinstatement of extinguished cocaine-seeking behaviors in rodents. However, far less is known about the role for hypocretin transmission in regulating ongoing cocaine-taking behavior. Here, we investigated the effects of the selective Hcrt-R1 antagonist SB-334867 on cocaine intake, as measured by intravenous (IV) cocaine self-administration in rats. The stimulatory effects of cocaine on brain reward systems contribute to the establishment and maintenance of cocaine-taking behaviors. Therefore, we also assessed the effects of SB-334867 on the reward-enhancing properties of cocaine, as measured by cocaine-induced lowering of intracranial self-stimulation (ICSS) thresholds. Finally, to definitively establish a role for Hcrt-R1 in regulating cocaine intake, we assessed IV cocaine self-administration in Hcrt-R1 knockout mice. We found that SB-334867 (1–4 mg/kg) dose-dependently decreased cocaine (0.5 mg/kg/infusion) self-administration in rats but did not alter responding for food rewards under the same schedule of reinforcement. This suggests that SB-334867 decreased cocaine reinforcement without negatively impacting operant performance. SB-334867 (1–4 mg/kg) also dose-dependently attenuated the stimulatory effects of cocaine (10 mg/kg) on brain reward systems, as measured by reversal of cocaine-induced lowering of ICSS thresholds in rats. Finally, we found that Hcrt-R1 knockout mice self-administered far less cocaine than wildtype mice across the entire dose-response function. These data demonstrate that Hcrt-R1 play an important role in regulating the reinforcing and reward-enhancing properties of cocaine and suggest that hypocretin transmission is likely essential for establishing and maintaining the cocaine habit in human addicts.

## Introduction

Cocaine dependence in humans is characterized by persistent drug use despite negative consequences and high rates of relapse to drug-taking behavior following intermittent periods of abstinence (Dackis and O'Brien, 2001). Hypocretin-1 (Hcrt-1) and hypocretin-2 (Hcrt-2), also known as orexin-A (OX_A_) and orexin-B (OX_B_), are lateral hypothalamic (LH) neuropeptides that have garnered much attention since their discovery in the late 1990s (Gautvik et al., 1996; De Lecea et al., 1998; Sakurai et al., 1998). While originally considered important regulators of metabolic, circadian, and stress systems (Lubkin and Stricker-Krongrad, 1998; Van Den Pol et al., 2001; Szekely, 2006), hypocretin transmission is emerging as a key signaling mechanism in relapse to drug-seeking during periods of abstinence (Dileone et al., 2003; Harris et al., 2005; Aston-Jones et al., 2009; Borgland et al., 2010). Hypocretin fibers innervate, and there are dense concentrations of hypocretin receptors, in brain regions implicated in drug reinforcement processes, such as the nucleus accumbens (Acb), ventral tegmental area (VTA), and bed nucleus of the stria terminalis (BNST) (Peyron et al., 1998; Nambu et al., 1999; Baldo et al., 2003). Moreover, the hypocretin-1 receptor (Hcrt-R1) antagonist, SB-334867, has been shown to attenuate stress- and cue-induced reinstatement of previously extinguished cocaine-, morphine-, and alcohol-seeking behavior (Boutrel et al., 2005; Harris et al., 2005; Lawrence et al., 2006; Kenny, 2011). These findings support the notion that Hcrt-R1 antagonists may have therapeutic utility for preventing relapse to cocaine use during periods of abstinence.

While Hcrt-R1 play a role in reinstating drug-seeking behaviors, their role in regulating ongoing drug-taking behavior is much less clear. This is important to understand, as a role for Hcrt-R1 in the persistence of drug-taking behavior would suggest that Hcrt-R1-based therapeutic agents may have utility not just in reducing rates of relapse but also in cessation of intake and achieving abstinence. Aston-Jones and colleagues found that SB-334867 had no effects on cocaine self-administration when rats responded for the drug on a fixed-ratio 1 schedule (Smith et al., 2009). However, SB-334867 significantly decreased cocaine self-administration behavior when rats were responding under more stringent progressive-ratio (PR) schedules of reinforcement (Borgland et al., 2009; Espana et al., 2010). Similarly, SB-334867 decreased cocaine intake in rats responding vigorously under a discrete-trial (DT) procedure that limits access to the drug to 10-min trials interspaced by 20-min intervals when drug access is withheld during 24-h long access sessions (Espana et al., 2010). These findings demonstrate that Hcrt-R1 may regulate ongoing cocaine intake, but that this effect occurs most prominently under reinforcement schedules that require high levels of effort and motivation to obtain the drug [for review, see Thompson and Borgland (2011)].

In addition to the schedule of reinforcement, the effects of SB-334867 on responding for cocaine in rats also depends upon a number of other factors, including food deprivation state of the animal, formulation of the compound (i.e., poor solubility), and the time of day (i.e., light/dark cycle) of testing. It is a notable concern that SB-334867 is derived from a chemical scaffold that generates compounds that can bind to non-Hcrt-R1 receptor targets including 5-HT_2b_ and 5-HT_2c_ receptors (Porter et al., 2001), which are relatively unstable *in vivo* with a short half-life and could therefore potentially yield bioactive metabolites. Further, the drug may have poor penetration in the central nervous system. Thus, it is possible that in addition to its effects on Hcrt-R1, SB-334867 could regulate cocaine intake through “off-target” actions at other classes of receptors in the brain. Considering that the vast majority of studies implicating Hcrt-R1 in addiction-related processes have utilized SB-334867, it is important to verify that the actions of the compound are directly related to antagonism of Hcrt-R1.

Here, we sought to determine the role for Hcrt-R1 in regulating ongoing cocaine self-administration behavior by first assessing the effects of SB-334867 on cocaine self-administration in rats. Next, we examined whether SB-334867 may modulate cocaine intake by impacting the reward-enhancing effects of cocaine, as measured by cocaine-induced lowering of intracranial self-stimulation (ICSS) thresholds in rats. Importantly, the reward-enhancing effects of cocaine are thought to provide a major source of motivation that contributes to the establishment and maintenance of the drug-taking habit (Kenny, 2007). Finally, to unambiguously test the role for Hcrt-R1 in regulating cocaine-taking behavior we assessed IV cocaine self-administration in mice with a null mutation of the Hcrt-1 receptor (Hcrt-R1^−/−^) in comparison with their heterozygous (Hcrt-R1^+/−^) and wildtype (Hcrt-R1^+/+^) littermates.

## Materials and methods

### Subjects

For the rat experiments, 24 male Wistar rats in total were used. Rats weighed 300–320 g upon arrival at the laboratory (Charles River Laboratories, Raleigh, NC, USA). In the mouse experiments, we obtained breeding pairs of Hcrt-R1^+/−^ mice backcrossed > 10 generations on a C57BL/6 mice background from Jackson Laboratory (Bar Harbor, ME). Mice for testing were obtained from crosses of heterozygous Hcrt-R1 male and female mice. For all experiments, rats and mice were housed in groups of 1–3 per cage, with food and water available *ad libitum*, in a temperature-controlled vivarium under a reversed 12-h light/dark cycle (lights off at 8 am). Animals were tested during the dark portion of the light/dark cycle between the hours of 11 am and 4 pm. All procedures were conducted in adherence with the National Institutes of Health Guide for the Care and Use of Laboratory Animals and were approved by the Institutional Animal Care and Use Committee of The Scripps Research Institute.

### Drugs

SB-334867 [N-(2-Methyl-6-benzoxazolyl)-N″-1,5-naphthyridin-4-yl urea hydrochloride] was purchased from Tocris Bioscience (MO, USA). Cocaine was supplied by the National Institute on Drug Abuse (NIDA). For IV self-administration, cocaine was dissolved in 0.9% (w/v) sterile saline, and IV infusions were earned by rats in a volume of 0.1 ml per 4-s infusion (0.5 mg/kg/infusion) or mice in a volume of 35.25 μl per 3-s infusion (0.3 mg/kg/infusion). For systemically administered cocaine injections in ICSS experiments, cocaine was dissolved in sterile saline and injected intraperitoneally (IP) in a volume of 1 ml/kg body weight. Cocaine was administered 10 min before ICSS experimental sessions. For systemic SB-334867 administration, SB-334867 was dissolved in 10:10:80, DMSO:tween 80:water (v:v:v) and delivered in a volume of 10 ml/kg body weight by IP injection 30 min before behavioral test sessions. Drugs were prepared freshly immediately before each administration.

### Surgery

Rats and mice to be prepared with IV catheters were anaesthetized by inhalation of 1–3% isoflurane in oxygen and silastic catheters were inserted into the jugular vein as described previously (Fowler et al., 2011). Briefly, the catheters consisted of a 14 cm (rat) or 6 cm (mouse) length of silastic tubing fitted to a guide cannula (Plastics one, Wallingford, CT), bent at a curved right angle and encased in dental acrylic. The catheter tubing was passed subcutaneously from the animals' back to the right jugular vein, and 1 inch (rat) or 1 cm length (mouse) of the catheter tip was inserted into the vein. After surgery, catheters were flushed daily with 0.1 ml of a heparinized (30 USP units/ml) sterile saline solution. Rats to be prepared with ICSS electrodes were anaesthetized by inhalation of 1–3% isoflurane in oxygen and positioned in a stereotaxic frame (Kopf Instruments, Tujunga, CA, USA). A stainless steel bipolar stimulating electrode (11 mm in length) was implanted into the posterior lateral hypothalamus (AP: −0.5 mm from bregma; ML: ±1.7 mm; DV: 8.3 mm from dura; incisor bar adjusted to 5 mm above the interaural line) (Pellegrino et al., 1979). Animals were allowed to recover from surgery for at least 7 days prior to training in the ICSS or self-administration procedures.

### Intravenous (IV) cocaine self-administration procedure

Mice and rats were mildly food restricted to 85–90% of their free-feeding body weight and trained to press a lever in an operant chamber (Med Associates, St. Albans, VT) for food pellets (20 mg food pellets, mice; 45 mg food pellets, rats; TestDiet, Richmond, IN) under a fixed-ratio 5, time out 20-s (FR5TO20 s) schedule of reinforcement prior to catheter implantation. Once stable responding was achieved (> 25 pellets per session in mice; > 90 pellets per session in rats), subjects were catheterized as described above. The animals were allowed at least 48 h to recover from surgery, and then permitted to respond for food reinforcement again under the FR5TO20 s schedule. Once food-responding criteria were re-established, subjects were permitted to acquire IV cocaine self-administration by autoshaping during 1-h daily sessions, 7 days per week. Cocaine was delivered through the tubing into the IV catheter by a Razel syringe pump (Med Associates). Each cocaine self-administration session was performed using 2 retractable levers (1 active; 1 inactive). Completion of the response criteria on the active lever resulted in the delivery of an IV cocaine infusion (0.03 ml infusion volume for mice; 0.1 ml for rats). Unit doses of cocaine contingently delivered upon completion of schedule requirements were 0.5 mg/kg/infusion for rat and 0.3 mg/kg/infusion for mouse. Delivery of a cocaine infusion coincided with the initiation of a 20-s time-out (TO) period, signaled by a light cue located above the lever. During the TO period, responding on the lever was recorded but without scheduled consequence. Responding on the inactive lever was recorded but also was without scheduled consequence throughout the session.

### Intracranial self-stimulation reward threshold procedure

Rats (*n* = 10) were food restricted to maintain body weight ~85% of free-feeding body weight and trained to respond according to a modification of a DT current threshold procedure (Kornetsky and Esposito, 1979; Markou and Koob, 1992). Briefly, a trial was initiated by the delivery of a non-contingent electrical stimulus. This electrical reinforcer had a train duration of 500 ms and consisted of 0.1 ms square wave pulses that were delivered at a frequency of 50–100 Hz. The frequency of the stimulation was selected for individual animals so that current-intensity thresholds (see below) of each subject were within 50–300 μA, and thus allowed both threshold elevation and lowering to be detected. This frequency for each rat was held constant throughout the experiment. A one-quarter turn of the wheel manipulandum within 7.5 s of the delivery of the non-contingent electrical stimulation resulted in the delivery of an electrical stimulus identical in all parameters to the non-contingent stimulus that initiated the trial. After a variable inter-trial interval (7.5–12.5 s, average of 10 s), another trial was initiated with the delivery of a non-contingent electrical stimulus. Failure to respond to the non-contingent stimulus within 7.5 s resulted in onset of the inter-trial interval. Responding during the inter-trial interval reset the inter-trial interval and thereby delayed the onset of the next trial. Current levels were varied in alternating descending and ascending series. A set of five trials was presented for each current intensity. Current intensities were altered in 5 μA steps. In each testing session, four alternating descending and ascending series were presented. The threshold for each series was defined as the midpoint between two consecutive current intensities that yielded “positive scores” (animals responded for at least three of the five trials) and two consecutive current intensities that yielded “negative scores” (animals did not respond for three or more of the five trials). The overall threshold of the session was defined as the mean of the thresholds for the four individual series. Each testing session was approximately 45 min in duration. The time between the onset of the non-contingent stimulus and a positive response was recorded as the response latency. The response latency for each test session was defined as the mean response latency of all trials during which a positive response occurred. After establishment of stable ICSS reward thresholds (defined as ≤10% variation in thresholds over a 3-day period), rats were tested in the ICSS procedure once daily.

### Experimental procedures

#### Cocaine self-administration in rats

Rats and mice were prepared with IV catheters and trained to respond for cocaine infusions as described above. Rats (*n* = 7) responding stably for cocaine infusions under a FR5TO20 s schedule of reinforcement were injected with SB-334867 (0, 1, 2, or 4 mg/kg; 30-min pre-treatment) according to a within-subjects Latin-square design. A minimum of 48 h was allowed between each SB-334867 injection, during which rats had their daily cocaine self-administration session, to ensure that rates of responding for cocaine returned to pre-injection baseline before the next SB-334867 administration. A second group of rats (*n* = 7) responding for food rewards under the same reinforcement schedule were injected with SB-334867 (0–4 mg/kg; 30-min pre-treatment) according to a within-subjects Latin-square design, and food intake was assessed.

#### Intracranial self-stimulation in rats

The dose of cocaine (10 mg/kg) used in the present studies was chosen based on previous observations that this dose induced maximal threshold lowering without affecting performance in the ICSS procedure used in the present study (Kenny et al., 2003) and was equivalent to the amount of cocaine consumed by self-administering rats during their daily 1-h access to cocaine self-administration. Rats (*n* = 10) were prepared with ICSS electrodes as described above and trained in the ICSS procedure until stable thresholds were achieved (≤10% variation in thresholds over 5 consecutive days). To determine if SB-334867 attenuated the magnitude of cocaine-induced lowering of ICSS thresholds, rats were injected with SB-334867 (0, 1, 2, 4, mg/kg; 30-min pre-treatment) according to a within-subjects Latin-square design. All rats then received a cocaine injection 20 min later, 10 min prior to initiation of the ICSS session. After this treatment regimen, rats were once again injected with SB-334867 (0, 1, 2, 4, mg/kg; 30-min pre-treatment) according to a within-subjects Latin-square design, but this time the injection given 10 min before the ICSS session was saline instead of cocaine.

#### Cocaine self-administration in mice

Hcrt-R1 wildtype, heterozygous, and homozygous knockout mice were trained to respond for food rewards, prepared with IV catheters, and permitted to respond for cocaine infusions (0.3 mg/kg/infusion; training dose) as described above. After mice established stable responding for the training dose, the unit dose available for consumption was varied to generate a dose-response function. All mice responded for each unit dose of cocaine in the dose-response curve for at least 5 days and were returned to the training dose for at least three successive days until stable levels of intake at that dose were again established between the different doses of cocaine tested.

### Statistical analyses

The effects of SB-336967 on cocaine intake and responding for food reinforcers in rats were analyzed by one-factor repeated-measures analysis of variance (ANOVA) with SB334867 dose (0–4 mg/kg) as the within-subjects repeated-measures factor. For the ICSS experiment, percentage change from baseline reward threshold was calculated by expressing the drug-influenced absolute threshold scores as a percentage of the baseline thresholds defined as the mean of the thresholds obtained on the 3 days before the first SB-334867 injection. Next, the percentage of baseline scores were subjected to two-factor repeated-measures ANOVA, with SB-334867 (0–4 mg/kg) and cocaine (0 or 10 mg/kg) as the two within-subjects factors. Response latency data were analyzed in the same manner as the threshold data. For the mouse cocaine self-administration experiment, data were analyzed by two-factor repeated-measures ANOVA with cocaine dose as the within-subjects factor and genotype as the between-subjects factor. Similarly, the food responding data in mice were analyzed by one-factor repeated-measures ANOVA with genotype as the between-subjects factor. Significant main or interaction effects were followed by Bonforroni *post-tests* or Newman–Keuls *post-hoc* tests as appropriate. All statistical analyses were performed using Graphpad Prism software and the level of significance was set at 0.05.

## Results

### Hcrt-R1 regulates cocaine self-administration behavior in rats

**Figure 1:**
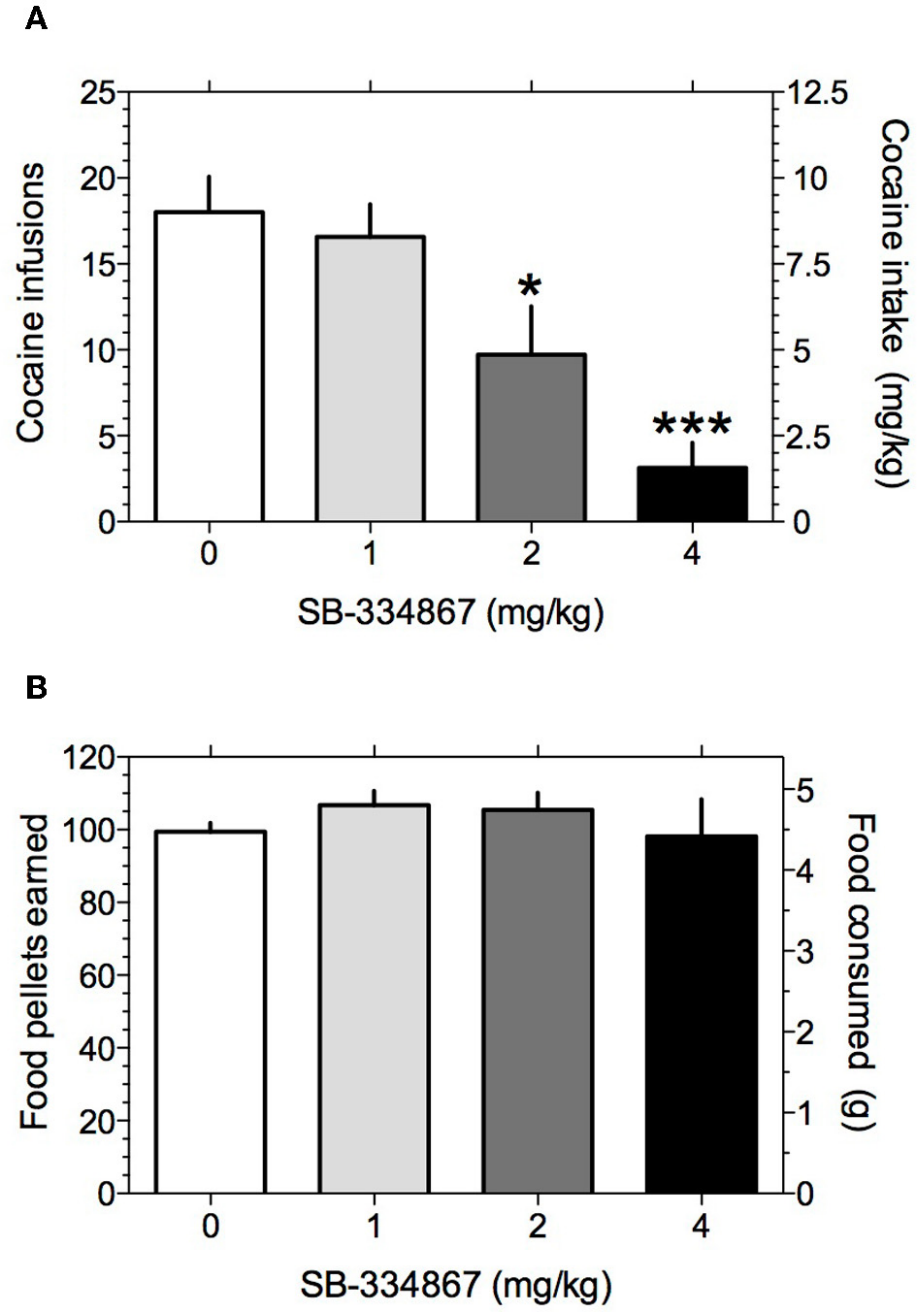
**Hypocretin transmission at Hcrt-1 receptors regulates cocaine intake.** The effects of systemically administered SB-334867 on responding for cocaine or food rewards were tested under a FR5TO20 s reinforcement schedule (see Materials and Methods). **(A)** Mean (± S.E.M.) number of cocaine reinforcers earned after treatment with the selective Hcrt-R1 antagonist SB-334867. ^*^P < 0.05 and ^***^P < 0.001 compared with vehicle treatment; *post-hoc* test after a significant main effect in One-Way repeated-measures ANOVA. **(B)** Mean (± S.E.M.) number of food reinforcers earned after treatment with SB-334867.

We first investigated the role of hypocretin transmission at Hcrt-R1 in regulating cocaine self-administration behavior in rats. Rats responding for IV cocaine infusions (0.5 mg/kg/infusion) under a FR5TO20 s schedule of reinforcement were treated with the selective Hcrt-R1 antagonist SB-334867 (0, 1, 2, and 4 mg/kg IP) and cocaine intake measured. To identify possible non-specific actions of SB-334867 on operant performance, we also assessed the effects of the drug on responding for food pellets in food restricted rats tested under the same reinforcement schedule. The mean number of food or cocaine rewards earned prior to treatment with SB-334867 was 94.3 ± 4.6 and 22.1 ± 1.9, respectively. One-Way repeated-measures ANOVA on the cocaine intake data following SB-334867 treatment revealed that SB-334867 significantly altered cocaine self-administration [*F*_(3, 27)_ = 14.5, *P* < 0.0001]. Bonferroni *post-tests* among means revealed that cocaine intake was significantly reduced at the 2 mg/kg (*P* < 0.05) and 4 mg/kg (*P* < 0.001) compared with vehicle treatment (Figure 1A). In contrast, One-Way repeated-measures ANOVA on the food intake data demonstrated no statistically significant effects of SB-334867 at any dose tested [*F*_(3, 27)_ = 0.6, *P* > 0.05; Figure 1B].

### Hcrt-R1 regulate the reward-enhancing effects of cocaine

**Figure 2:**
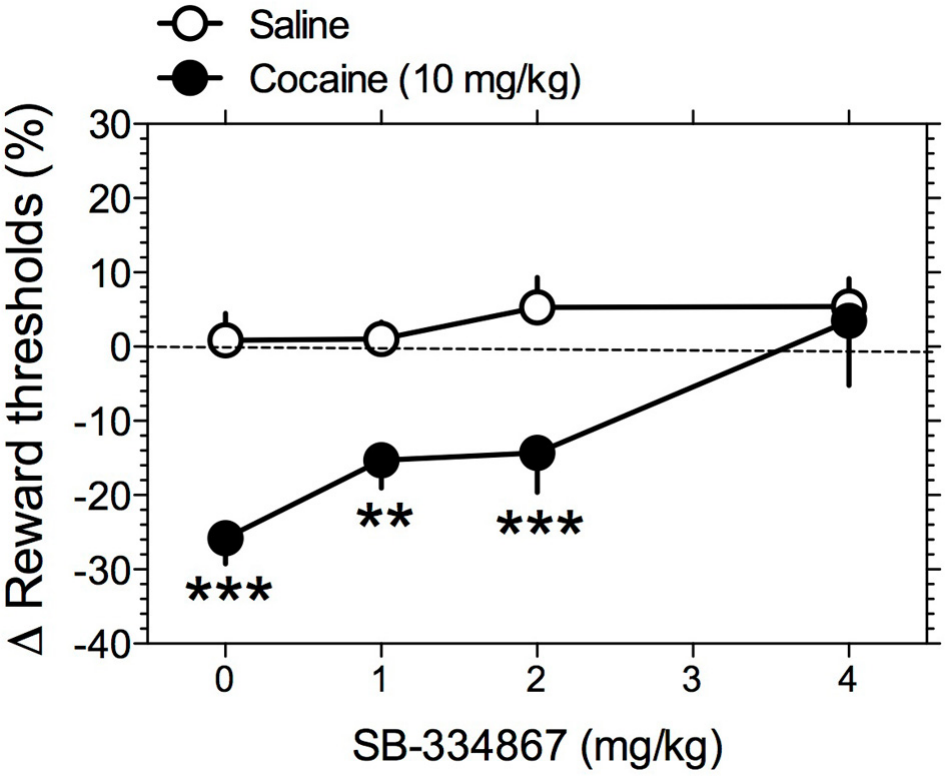
**Hypocretin transmission at Hcrt-1 receptors regulates the reward-enhancing effects of cocaine.** The effects of SB-334867 on cocaine-induced lowering of ICSS thresholds were tested. Rats were pre-treated with SB-334867 (0, 1, 2, or 4 mg/kg), and subsequently received saline or cocaine (10 mg/kg) injections. Data are expressed as mean (± S.E.M.) percentage change from baseline thresholds. ^**^P < 0.01 and ^***^P < 0.001 compared with the saline pre-treatment at the same dose of SB-334867; *post-hoc* comparisons after significant main effect of Cocaine and interaction effect with SB-334867 in Two-Way repeated-measures ANOVA.

Similar to other drugs of abuse, cocaine enhances the activity of the brain reward systems, as measured by lowering of ICSS thresholds (Esposito et al., 1978; Maldonado-Irizarry et al., 1994). One mechanism by which SB-334867 could decrease cocaine intake in rats is by attenuating the stimulatory effects of cocaine on brain reward systems. To test this hypothesis, we assessed the effects of SB-334867 (0, 1, 2, and 4 mg/kg IP) on cocaine-induced lowering of ICSS thresholds in rats. Mean absolute ICSS thresholds prior to SB-334867 treatment was 115.5 ± 10.5 μA. Two-Way repeated-measures ANOVA demonstrated a significant main effect of cocaine [*F*_(1, 16)_ = 83.09, *P* < 0.0001] and significant SB-334867 X cocaine interaction [*F*_(3, 16)_ = 8.61, *P* < 0.01], but no effect of SB-334867 [*F*_(3, 16)_ = 2.62, *P* > 0.05]. Pre-planned comparisons among means demonstrated that reward thresholds were significantly lowered by vehicle-cocaine (*P* < 0.0001) treatment compared with vehicle-saline treatment. Pre-treatment with the lowest doses of SB-334867 (0–2 mg/kg) did not alter the threshold-lowering effects of cocaine compared with saline treatment (Figure 2). However, pre-treatment with the highest dose of SB-334867 (4 mg/kg) completely blocked this effect of cocaine. Response latencies were unaltered by any treatment condition (data not shown).

### Hcrt-1 knockout mice are less sensitive to cocaine reinforcement

**Figure 3:**
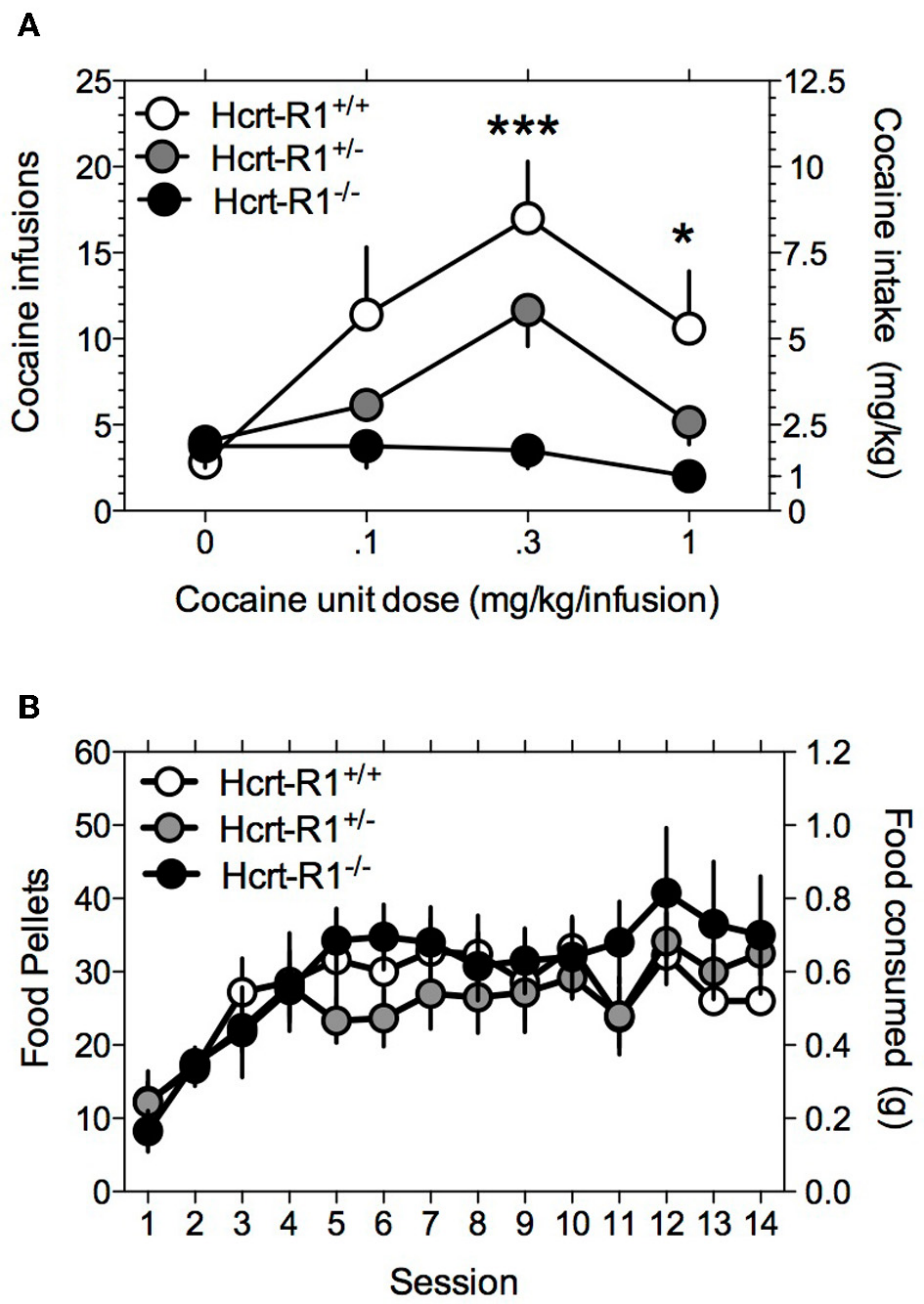
**Genetic deletion of Hcrt-1 receptors greatly diminishes the reinforcing properties of cocaine.** Responding for cocaine and food reward was tested in Hcrt-R1^−/−^, Hcrt-R1^+/−^, and Hcrt-R1^+/+^ mice. Data is expressed as mean (± S.E.M.) number of cocaine **(A)** or food (**B**) reinforcers earned on a FR5TO20 schedule of reinforcement. ^*^P < 0.05 and ^***^P < 0.001 compared Hcrt-R1^+/+^ to Hcrt-R1^−/−^ mice; *post-hoc* comparisons made after Two-Way repeated-measures ANOVA calculated.

Although the Hcrt-R1 antagonist SB-334867 is 50-fold more selective for Hcrt-R1 over the Hcrt-R2 receptor (Smart et al., 2001), as with all other pharmacological probes there is the possibility that it may act at other receptor targets (Porter et al., 2001). Thus, to verify that the Hcrt-R1 indeed regulates ongoing cocaine-taking behavior, we assessed cocaine self-administration in Hcrt-R1^−/−^, Hcrt-R1^+/−^, and Hcrt-R1^+/+^ mice. A Two-Way repeated-measures ANOVA revealed significant main effects for genotype [*F*_(2, 36)_ = 4.2, *P* < 0.05] and cocaine dose [*F*_(3, 36)_ = 12.9, *P < 0.0001*], with a significant genotype X cocaine dose interaction [*F*_(6, 36)_ = 4.3, *P* < 0.01]. Bonferroni *post-tests* among means revealed that cocaine intake was significantly reduced in the Hcrt-1^−/−^ compared to the Hcrt-1^+/+^ mice at the 0.3 mg/kg (*P* < 0.001) and 1 mg/kg (*P* < 0.05) cocaine doses (Figure 3A). One-Way repeated-measures ANOVA on the food intake data demonstrated no statistically significant effects of genotype across the 14 days of food self-administration [*F*_(2, 152)_ = 2.79, *P* > 0.05; Figure 3B].

## Discussion

Our findings support the growing literature showing that hypocretin is a critical signaling neuropeptide that regulates the motivational and psychomotor stimulant properties of cocaine and other drugs of abuse. Previous work has shown that blockade of Hcrt-R1 transmission decreases locomotor sensitization to cocaine (Borgland et al., 2006) and attenuates the reinstatement of previously extinguished responding for the drug (Boutrel et al., 2005; Smith et al., 2009; James et al., 2011). Here, we show that the selective Hcrt-R1 antagonist SB-334867 decreases cocaine (but not food) self-administration in rats. Further, we found that SB-334867 dose-dependently blocked the stimulatory effects of cocaine on brain-reward circuitry, as measured by ICSS thresholds. Finally, we show for the first time that mice lacking functional Hcrt-R1 (Hcrt-R1^−/−^) exhibit greatly diminished responsiveness to the self-administration of cocaine. Collectively, these findings suggest that Hcrt-1 signaling is essential for maintaining ongoing cocaine-taking behavior and may be a promising target in the development of pharmacotherapies to treat cocaine dependence.

Our findings further show that blockade of Hcrt-R1 transmission using the selective antagonist SB-334867 dose-dependently decreased cocaine intake in rats at doses that did not alter responding for food reinforcers. Importantly, previous work from our laboratory has shown that the low dose of SB-334867 used in this study (4 mg/kg; IP) was still detectable in brain tissues and blood 2 h following administration (Hollander et al., 2008). Our rationale for selecting low doses of SB-334867 in our studies is based on evidence that doses greater than 10 mg/kg can produce disturbances in sleep/awake cycles (Smith et al., 2003) as well as a number of active behaviors (feeding, grooming, sniffing, locomotion, and rearing) (Rodgers et al., 2001). Nevertheless, our findings suggest that Hcrt-R1 play an essential role in maintaining ongoing cocaine intake and highlight the possibility that novel Hcrt-R1 antagonists suitable for use in humans may have therapeutic utility for the treatment of cocaine dependence. It is important to note that previous studies have provided mixed results with regard to the role for Hcrt-R1 in cocaine reinforcement. Aston-Jones and colleagues have shown that SB-334867 does not decrease cocaine intake in rats responding under a FR1 schedule of reinforcement (Smith et al., 2009). In contrast, SB-334867 decreased cocaine intake in rats responding under schedules requiring greater degrees of effort and motivation to obtain the drug (PR and DT reinforcement schedules; see Introduction) (Borgland et al., 2009; Espana et al., 2010). In our study, rats responded under a FR5 schedule, which requires greater levels of effort to obtain the drug than FR1 schedules. While we did not manipulate the reinforcement schedule in our experiments, we speculate that SB-334867 and genetic knockout of the Hcrt-R1 are most effective in reducing cocaine self-administration when effort requirements are high. For example, we plan to test in future studies the hypothesis that Hcrt-R1^−/−^ may show more similar levels of cocaine intake to Hcrt-R1^+/+^ under an FR1 schedule, but would exhibit decreased responding as the effort to obtain cocaine is increased (e.g., FR-5, PR). Nevertheless, our findings support a key role for Hcrt-R1 in cocaine reinforcement, and further support the notion that the effects of Hcrt-R1 transmission in this process are dependent on the degree of motivation required to seek and obtain the drug.

With extensive evidence demonstrating the importance of hypocretin in arousal state (Moore et al., 2001; Yoshimichi et al., 2001; Sasaki et al., 2011; Tsunematsu et al., 2011), it is possible that the decreased effects of cocaine we observed following the blockade of Hcrt-R1 signaling is due to a generalized disruption of stress systems. Certainly, there is a strong link between hypocretin functioning and biochemical modulation of stress pathways in the CNS. For example, it has been shown that the non-selective corticotropin-releasing factor (CRF) receptor antagonist α-helical CRF blocked hypocretin-induced grooming and face-washing behaviors in rats (Ida et al., 2000). Further, administration of the Hcrt-1 peptide dose-dependently increased circulating corticosterone levels and central expression of CRF mRNA (Ida et al., 2000; Al-Barazanji et al., 2001; Russell et al., 2001). Additionally, Boutrel and colleagues found that ICV administration of the Hcrt-1 peptide elevated ICSS thresholds, which indicates a decrease in the activity of brain reward systems (Boutrel et al., 2005). Based on these observations, it is plausible that hypocretins regulate the reinforcing effects of cocaine by neural circuitry associated with stress and arousal-related processes. However, as shown here and in a prior report from our laboratory (Hollander et al., 2008), SB-334867 administration alone did not elevate ICSS thresholds in rats, an increase that is indicative of dysphoria or an aversive state (Carlezon and Chartoff, 2007), and which may be expected in the case of increased CRF signaling (Macey et al., 2000; Bruijnzeel et al., 2009). While there are reports that show Hcrt-R1^−/−^ mice have fragmented sleep/wake cycles (Willie et al., 2003; Mieda et al., 2011), many groups have found that these animals do not appear to have an overt arousal/stress phenotype or behavioral abnormalities (Hungs and Mignot, 2001; Willie et al., 2001; Sakurai, 2007; Hondo et al., 2010). Therefore, our data support the notion that Hcrt-R1 have a critical role in cocaine reinforcement that, at least in part, can act independent of endogenous stress systems.

The ICSS procedure used here is based on the premise that electrical self-stimulation of certain brain areas (e.g., lateral hypothalamus, VTA) is highly rewarding and may be a useful strategy when assessing changes in the motivational influences of cocaine (Kornetsky and Esposito, 1979; Fish et al., 2010). In our studies, the ICSS procedure gives a significant advantage over the self-administration experiments in that it is possible to obtain a physiological measure of the stimulatory effects of cocaine on brain reward systems. The ICSS reward threshold is defined as the minimum electrical current necessary to support self-stimulation behavior by the animal and is remarkably consistent across test sessions. Thus, the ICSS procedure is considered an accurate measure of how well brain reward systems are functioning. Importantly, acute administration of cocaine and all other major drugs of abuse have been shown to lower the ICSS threshold (Markou and Koob, 1992; Bespalov et al., 1994; Harrison et al., 2002), while drug withdrawal diminishes brain reward function and elevates ICSS thresholds (Ahmed et al., 2002; Kenny and Markou, 2005; Bruijnzeel et al., 2007). Obtaining the reward-enhancing properties of cocaine, reflected in its lowering effects on ICSS thresholds (Esposito et al., 1978; Maldonado-Irizarry et al., 1994), is thought to provide an important source of motivation that contributes to the establishment and maintenance of the cocaine-taking habit (Kenny, 2007). The cocaine-induced lowering of the ICSS threshold was dose-dependently blocked with the Hcrt-1 antagonist, suggesting that SB-334867 reduces the reward-enhancing effects of cocaine. These results are reminiscent of recent findings from our laboratory showing that similar doses of SB-334867 abolished nicotine-induced lowering of ICSS thresholds in rats (Hollander et al., 2008). However, this result appears to conflict with a recent report by Riday and colleagues who found that SB-334867 had no effects on the cocaine-enhanced responding for ICSS in Swiss-Webster mice (Riday et al., 2012). Importantly, mice responded for ICSS in this prior study under a continuous reinforcement schedule (FR1), which requires low levels of effort to obtain rewarding ICSS (Riday et al., 2012). In contrast, responding for ICSS in our study occurred under a DT current-threshold procedure that requires greater degrees of effort and motivation to obtain stimulation compared with FR1 schedules. As noted above, SB-334867 does not appear to decrease cocaine intake in rats responding under a FR1 reinforcement schedule (Smith et al., 2009), but decreases intake in rats responding under schedules requiring higher levels of motivation (Borgland et al., 2009; Espana et al., 2010). Hence, the apparent discrepancy between our current study and that of Riday and colleagues likely reflects the levels of effort required to obtain ICSS in both studies.

We found that heterozygous or homozygous for null mutation in Hcrt-R1 demonstrated a gene-dosage-type decrease in responding for cocaine across the entire dose-response function, typically interpreted as a decrease in motivation to consume cocaine. Importantly, these mice did not show any deficit in responding for food reinforcers under the same reinforcement schedule. In fact, there was a trend for the Hcrt-R1^+/−^ and Hcrt-R1^−/−^ to responding at higher rates for food reinforcers. This verifies that the decreased responding for cocaine seen in these mice was not secondary to disruption in learning- or performance-related processes that could indirectly have resulted in lower levels of lever-pressing for cocaine. These findings provide definitive evidence that Hcrt-R1 is essential for the reinforcing properties of cocaine. A distinct disadvantage of using Hcrt-1 knockout mice in our study is the possibility that the behavioral effects observed are due to genetic compensation mechanisms, such as altered expression of Hcrt-2 receptor signaling in these animals. Intriguingly, both hypocretin receptor subtypes are concentrated in a number of brain areas important for reward-related processing, including the VTA, accumbens, BNST, and CeA (Peyron et al., 1998; Nambu et al., 1999; Narita et al., 2006). However, to date, there has been very little evidence suggesting a prominent role for Hcrt-2 transmission in mediating the rewarding properties of drugs of abuse such as cocaine. For example, VTA perfusion of Hcrt-1 (but not Hcrt-2) was found to increase VTA release of glutamate and dopamine, as well as successfully reinstate extinguished cocaine seeking (Wang et al., 2009). While transgenic mice with both receptors knocked out showed reduced dopaminergic responses to cocaine (Espana et al., 2010), the Hcrt-R1 antagonist SB334867 was found to block the effects of cocaine on glutamatergic plasticity of VTA dopamine neurons (Borgland et al., 2006). In addition, administration of the Hcrt-2 receptor antagonist 4-pyridylmethyl (*S*)-*tert*-leucyl 6,7-dimethoxy-1,2,3,4-tetrahydroisoquinoline (4PT) had no effect on cocaine intake and cue-induced reinstatement in rats (Smith et al., 2009). Furthermore, while repeated cocaine injections in rats showed upregulated Hcrt-2 protein levels in the accumbens, this effect was not found in several other important reward-related limbic structures including the frontal cortex, hippocampus, VTA, and caudate putamen (Zhang et al., 2007). Although these observations suggest that alterations in Hcrt-2 receptors are unlikely to account for our findings, other compensatory mechanisms independent of Hcrt-1 signaling could potentially contribute to the deficits in drug intake in the heterozygous and knockout mice. To account for this possibility, future investigations will need to evaluate cocaine self-administration in mice with conditional deletion of Hcrt-R1 in a spatially and temporally controlled fashion, or animals where virus is used to re-express the missing Hcrt-1 gene in targeted brain sites.

In sum, our findings suggest that hypocretin transmission at Hcrt-R1 may be a critical signaling cascade in the rewarding and motivational properties of cocaine. As such, there may be a tremendous potential therapeutic utility of Hcrt-R1 antagonists in the treatment of cocaine dependency in addicts who wish to remain drug-free.

### Conflict of interest statement

The authors declare that the research was conducted in the absence of any commercial or financial relationships that could be construed as a potential conflict of interest.
